# Three discipline collaborative radiation therapy (3DCRT) special debate: The United States needs at least one carbon ion facility

**DOI:** 10.1002/acm2.12727

**Published:** 2019-10-01

**Authors:** Eleanor A Blakely, Bruce Faddegon, Christopher Tinkle, Charles Bloch, Michael Dominello, Robert J Griffin, Michael C Joiner, Jay Burmeister

**Affiliations:** ^1^ Lawrence Berkeley National Laboratory Berkeley CA USA; ^2^ Department of Radiation Oncology University of California – San Francisco San Francisco CA USA; ^3^ Department of Radiation Oncology St. Jude Children’s Research Hospital Memphis TN USA; ^4^ Department of Radiation Oncology University of Washington Seattle WA USA; ^5^ Department of Oncology Wayne State University School of Medicine Detroit MI USA; ^6^ Department of Oncology University of Arkansas for Medical Sciences Little Rock AR USA; ^7^ Gershenson Radiation Oncology Center Barbara Ann Karmanos Cancer Institute Detroit MI USA

## 1 THREE DISCIPLINE COLLABORATIVE RADIATION THERAPY (3DCRT) DEBATE SERIES

Radiation oncology is a highly multidisciplinary medical specialty, drawing significantly from three scientific disciplines — medicine, physics, and biology. As a result, discussion of controversies or changes in practice within radiation oncology involves input from all three disciplines. For this reason, significant effort has been expended recently to foster collaborative multidisciplinary research in radiation oncology, with substantial demonstrated benefit.(1, 2) In light of these results, we endeavor here to adopt this “team‐science” approach to the traditional debates featured in this journal. This article is part of a series of special debates entitled “Three Discipline Collaborative Radiation Therapy (3DCRT)” in which each debate team will include a radiation oncologist, medical physicist, and radiobiologist. We hope that this format will not only be engaging for the readership but will also foster further collaboration in the science and clinical practice of radiation oncology.

## 2 INTRODUCTION

High linear energy transfer (LET) radiotherapy has long held the promise of improved efficacy against tumors that are refractory to conventional radiotherapy. While such an increase in efficacy is achievable with fast neutron therapy, poor physical dose distribution characteristics have limited its potential. Carbon ions are an elegant solution in that they combine the biological effectiveness of fast neutron therapy with physical dose shaping characteristics even better than proton therapy. However, the cost of technology to deliver this treatment is tremendous and its clinical potential remains largely unproven. Clinical trials are underway in several countries, helping us gather the necessary data to demonstrate its efficacy. However, the United States, a traditional world leader in the development and implementation of advanced healthcare technology, is not among them. Considering the substantial potential benefit of this treatment and also our current efforts to contain the costs of healthcare, is now the time for the United States to step from the sidelines to participate in this research? This is the subject of this month’s 3DCRT debate.

Arguing for the proposition will be Drs. Eleanor Blakely, Bruce Faddegon, and Christopher Tinkle. Dr. Blakely is a senior staff in biophysicist (rehired retiree since 2015) at the Lawrence Berkeley National Laboratory (LBNL) with more than 44 yr of professional experience in molecular, cellular, and animal radiobiological research directed at studying the basic mechanisms of radiation responses, with an emphasis on charged particle radiation effects. Dr. Faddegon is a professor of medical physics in the radiotherapy department of UCSF. His research focus is to bring technical innovation into the clinic to improve radiotherapy by advancing linear accelerator, imaging, and particle therapy equipment and methods including Monte Carlo simulation tools and techniques. Dr. Tinkle is a radiation oncologist and Assistant Member in the department of radiation oncology at St. Jude Children’s Research Hospital. His focus is on preclinical and translational studies in pediatric cancers exploring the interactions of proton and photon radiotherapy and emerging targeted systemic therapy.

Arguing against the proposition will be Drs. Charles Bloch, Michael Dominello, and Robert Griffin. Dr. Bloch is a medical physicist who started his career in proton therapy over 25 yr ago. Currently, he is an associate professor in the department of radiation oncology at the University of Washington and serves as an associate director of education, research, and development at the Seattle Proton Therapy Center. Dr. Dominello is an assistant professor in oncology department and practicing radiation oncologist at Wayne State University, Karmanos Cancer Center. His interests include stereotactic radiosurgery for brain and spine and therapeutic ratio. He currently serves as the Karmanos/McLaren‐wide PI for NRG, Karmanos Cancer Network Medical Director for Quality and participates as a member of numerous committees through NRG and ASTRO. Dr. Dominello serves as the Assistant Program Director for the Radiation Oncology Residency Program and as an instructor in courses for both graduate and undergraduate students at the university. Dr. Griffin is a professor of radiation biology at the University of Arkansas for Medical Sciences. His group studies living tissue response to high‐dose radiotherapy (SBRT) and spatially fractionated radiation approaches with targeted drug delivery to tumors. He served as a president of the Society for Thermal Medicine, is a Vice Chair of the Science Education and Professional Development Committee and Annual Meeting biology track chair for ASTRO and is an associate senior editor for Technology in Cancer Research and Treatment and the International Journal of Radiation Oncology, Biology and Physics.

## 3 OPENING STATEMENTS

### 3.1 Eleanor Blakely, PhD; Bruce Faddegon, PhD; Christopher Tinkle, MD, PhD

Ion therapy was pioneered at Lawrence Berkeley National Laboratory (LBNL)^3^, ^4^ to investigate the clinical value of improved dose sparing, enhanced relative biological effectiveness (RBE), and lower oxygen enhancement ratio (OER) by using this form of radiotherapy. Despite the origins of carbon ion radiotherapy (CIRT) in the United States, initial phase I/II studies done in conjunction with the University of California, San Francisco (UCSF) were cut short because of the closure of the LBNL facility.^5^ Henceforth in the United States, clinical particle therapy has been restricted to proton therapy and we have had to rely on the experience of our colleagues outside the United States for clinical investigations of CIRT.

CIRT has not been adopted in the United States because of cost and the absence of high‐level clinical evidence.^6^, ^7^ A CIRT facility currently costs substantially more to build and run than a proton facility with the same number of gantries and fixed beam lines, and level I clinical data in support of CIRT is currently lacking. We argue here that the limited clinical data and the ongoing international work speak to the need for a US investment in at least one CIRT facility to definitively evaluate the potential advantages and opportunities for cost containment of this form of advanced radiotherapy for Americans.

### 3.2 Physics: Increased target conformation

There are significant physical advantages of CIRT over photon and proton treatments.^8^ Intensity modulated particle therapy (IMPT) uses scanned beams that conform more closely to the target with lower integral dose to healthy tissues due to a higher peak‐to‐plateau ratio along the Bragg curve and reduced lateral spread. Further advances promise to improve conformality,^9^, ^10^ including improved accuracy in the assessment of the stopping power distribution in individual patients through direct measurement with dual‐energy x‐ray CT or particle CT to mitigate range uncertainty, and image guidance at the time of treatment with x rays, particles, or secondaries (PET, prompt gamma, etc.).^11^ Financial barriers are being addressed through efforts to reduce accelerator size and the size and weight of gantries.

### 3.3 Radiobiology: Increased therapeutic ratio

Recent particle radiobiology studies indicate a number of potential underlying mechanisms for the promising phase I/II clinical outcomes with carbon ions.^12^, ^13^ The tracks of individual stopping particles in carbon ion‐targeted tumor tissue leave behind a non‐homogeneous pattern of ionization clusters of reactive oxygen species (ROS)‐induced molecular changes that elicit unique characteristics that may be advantageous compared to photons. Carbon ions can enhance tumor eradication by modifying proteins associated with tumor radioresistance. For example, CIRT may limit HIF‐1a stabilization, MMP‐2 expression, and/or activation of the main epithelial–mesenchymal transition (EMT) signaling pathways that can trigger migration/invasion under normoxic and hypoxic conditions prevalent in radioresistant tumors relative to x rays.^14^ Compared to x rays or protons, carbon ions have also been reported to be more effective in decreasing cell survival and migration in prostate and pediatric medulloblastoma cells, as well as inducing more significant alteration in Hedgehog genes, another metastasis signaling pathway.^15^ Future biologically optimized treatment planning may be critical to the successful implementation of these advantages.^16^, ^17^, ^18^, ^19^, ^20^ CIRT may induce enhanced immune responses in the tumor and host.^21^, ^22^, ^23^, ^24^, ^25^ Future implementation of emerging information on radiogenomics may also allow individual patient selection for CIRT.^26^

### 3.4 Clinical Oncology: Better outcomes

The authors acknowledge that although the current clinical evidence supporting the use of carbon ion therapy is compelling, it is incomplete.^5^, ^27^, ^28^ The pioneering investigations at LBNL showed that fractionated delivery of heavy ion therapy, generally as a boost at relatively low doses, was well tolerated across diverse tumor sites, including locally advanced prostate cancer, bone and soft tissue sarcomas, and head and neck tumors of the salivary gland and paranasal sinus.^4^, ^29^, ^30^, ^31^, ^32^, ^33^, ^34^, ^35^, ^36^, ^37^ Subsequent phase I/II trials conducted largely in Japan and Germany employing higher doses and conducted primarily solely with CIRT have demonstrated negligible toxicity and encouraging tumor control rates in diverse histologic tumor types, including adenocarcinoma, adenoid cystic carcinoma, malignant melanoma, and bone and soft tissue sarcomas, tumors which are often resistant to conventional photon irradiation.^38^, ^39^, ^40^, ^41^, ^42^, ^43^, ^44^, ^45^ Additional difficult to treat tumor sites with early favorable outcomes include tumors of the lung, liver, and pancreas. To more clearly address the clinical utility of this modality, randomized phase III trials are ongoing internationally in tumor sites as varied as the head and neck,^46^ skull base,^47^ and pancreas,^48^ the latter of which is led by the United States. The value of these trials is limited for American patients, however, as generalization of the trial results to American patients cannot be made for studies that do not include them, and there is no facility in the United States to conduct these trials.

### 3.5 Why do we need a carbon ion radiotherapy facility in the United States?

CIRT, compared with photons and protons, has the potential for superior dose distributions with enhanced biological effectiveness at a much lower cost than current facilities. Phase I/II trials suggest CIRT may well improve cancer outcomes. However, current high‐level phase III randomized clinical trial evidence does not exist.

At least one CIRT facility is needed in the United States to (1) establish dose and fractionation regimens for US patients in different disease sites that are expected to benefit from CIRT; (2) conduct randomized clinical trials to establish which sites benefit from CIRT for the endpoints of therapeutic ratio, tumor control, treatment complications, and quality of life; (3) employ the best technology for the highest achievable accuracy and precision in targeting; (4) establish cost reductions through technology (keep it small), amortization (decades), and patient load (reduced number of fractions); and (5) allow further investigations on the radiobiology of CIRT to elucidate tumor‐specific (epi)‐genomic determinants and tumor microenvironmental and immune‐modulatory effects that govern response and resistance.

This carbon facility would pave the way to establish high‐level evidence for CIRT and would facilitate the technological innovation required to bring the cost down, advance treatment planning solutions, and improve the accuracy and reproducibility of treatment delivery.

### 3.6 Charles Bloch, PhD; Michael Dominello, DO; Robert Griffin, PhD

Our consensus opinion about this topic is that, in light of the longstanding and ongoing struggles that many if not all proton centers have had, we are not in favor of the expansion of another ion beam therapy at great expense and questionable improvement in patient care. The following topics comprise the majority of the rationale and relevant literature in support of our assertion.

It has been hypothesized that among patients undergoing radiotherapy, approximately 14–15% would potentially benefit from the dosimetric advantages afforded by proton therapy.^49^ The number that would benefit from the added advantage (if there is one) of carbon would amount to a fraction of that same 14–15% of patients. This is inadequate to justify the cost of building one or more carbon facilities which will have to treat many other patients (e.g., prostate) to maintain financial viability while offering no clinical advantage to these other patients.^50^

Furthermore, despite acceptance of the potential advantage for protons in the select 14–15% of patients mentioned above, a number of proton centers have gone bankrupt and others struggle financially in part because they are not treating enough patients to meet their business model (See https://www.nytimes.com/2018/04/27/business/proton-therapy-finances.html). While a single carbon facility in the United States may be able to attract many patients due to its novelty — which in large part will not be based on scientific evidence related to decreased side effects, new biology, or any other rational reason to pursue carbon ion therapy — it is not a viable modality to expand on top of the proton facility network already in existence, and already struggling. An additional obstacle beyond inadequate patient numbers for proton centers is the very real dilemma of insurance reimbursement. Some insurers will not reimburse for certain sites (e.g., prostate and head and neck) while other centers get reimbursed at a rate that is not financially sustainable.^51^ If new carbon centers are built, therapy is not going to be cheaper, and we predict will be considered even more experimental by insurers (with less clinical evidence), and reimbursement will be an even larger issue. Essentially these realities alone argue very strongly against the viability of carbon ion therapy in the United States.

If any facilities were built, the logical thing would be to establish a single facility with the sole focus of carbon ion clinical research, to gather evidence to support (or refute) building more centers. However, single‐center clinical trials are suboptimal sources of evidence.^52^, ^53^ In addition, a single center is unlikely to have enough patients to provide strong clinical evidence or it will take a long time to gather enough valid results. For many years, the United States had only one or two proton centers and failed to provide convincing clinical evidence of their advantage. A single carbon facility is unlikely to make a big impact in terms of the clinical research that would be necessary to motivate patients, payers, and the medical community at large with regard to possible advantages of carbon over proton therapy or even state‐of‐the‐art photon therapy.

As a case in point, this is not necessarily a new topic that has never been considered for the United States. The United States had a heavy ion radiation therapy program at Lawrence Berkeley for many years between 1960s and 1990s.^54^, ^55^, ^56^ After decades, that program was closed without providing any strong support for construction of a clinical replacement. Other examples include Germany and Japan, who have carbon ion facilities that have been operating for decades, yet without providing proof of a significant clinical advantage in most contexts. The research and comparisons have been done and it is unconvincing as reported by a number of statistically scrutinized reports.^27^

The primary argument for carbon beam is enhanced RBE. However, RBE is something of a double‐edge sword. Neutron therapy was an early example of a modality that delivers an enhanced RBE (3 or more). However, for the most part, neutron treatments used lower physical dose (by a factor of 3 or more) so that the net effect was the same as a photon beam. Only a few targets (e.g., salivary gland 57, 58) were identified where the differential RBE made enough of a difference to offer a clinical advantage. However, those specific applications were not enough to economically or scientifically support any of the neutron facilities that were established and have all but gone away at present. There is no evidence that a carbon beam’s RBE would have more of a clinical advantage than neutrons and thus we are hard pressed to find any rationale for carbon ion center development.

Drilling down into the basic scientific rationale in terms of physics and biology, the hypothesis of the RBE advantage is that the RBE is highest in the Bragg peak, and therefore in the target and not in the entrance region.^59^ Therefore, the prediction with this knowledge considered in isolation is that there could be a strong clinical impact. Unfortunately, there are a number of problems with this argument. If you look at the clinical practice of existing carbon facilities,^38^ the most distal Bragg peak in a spread out Bragg peak (SOBP) is given a reduced weight, that is, less physical dose so that when combined with the expected RBE, the biological effect is the same as that of photons; 60 Gy (RBE) has the same effect as 60 Gy. So, there is no advantage of a higher RBE if you compensate using a lower dose (same as for neutrons). Second, all radiation therapy uses margins. This means the SOBP goes past the distal edge of the GTV, past the distal edge of the CTV, and into what is presumed to be normal tissue. That is, the particles with the highest RBE many times end up being deposited in normal tissue, not tumor. Several papers have identified this as a potential problem in proton therapy, specifically more brain necrosis than expected. 60, 61, 62 This may be due to the RBE in normal brain for protons being higher than 1.1, which is likely only more probable if using carbon ions and a SOBP.

After many years of research, the proton RBE has not changed much from the hypothesized value of 1.1 from Herman Suit.^63^ The best experimental value seems to be 1.1 ± 0.1, that is, effect is somewhere between non‐existent and twice what is used clinically. Due to the increased damage that will be inflicted by carbon in general, uncertainty in the clinical value of carbon RBE is likely to be even more clinically significant, thereby reducing how effective the modality can be in our opinion.

As it stands, there is a fundamental inability to predict the beam range as accurately as desired. This leads to increased margins. Treatment planning comparisons between charged particles and photons often show less normal dose due to the Bragg peak and lower integral dose.^64^, ^65^ However, those plans regularly assume the same target is used for both ion beams and photons. In reality, proton targets are larger due to the range uncertainty and this negates some of the potential benefit. Another proposed “advantage” of a carbon facility is the sharper Bragg peak even than protons. We submit that in most scenarios, the carbon Bragg peak is actually sharper than needed and considerable effort is then spent to expand the high‐dose region into a useable SOBP — more than is done with protons. This is accomplished using range modulators, energy layer stacking, or ridge filters. Compounding these realities of trying to deliver a quality treatment field is that the distal edge of the carbon beam is not nearly as “clean” as the proton Bragg peak and contains a tail (of unknown but presumably high RBE) beyond the end of the Bragg peak due to fragmentation of the carbon ions.^54^, ^66^

Another major concern is motion management, an issue that is critical for particle beams. Not only is there a moving target, but pencil‐beam scanning is delivering dose in a dynamic manner in three dimensions, two from the scanning system and one from the layer/range/energy changes. It has been reviewed how this may negatively affect daily dosing in a number of reports.^67^

The emerging technologies are immunotherapy, MR‐linac, radioligand therapy, and Flash radiotherapy. These are potentially game‐changing, disruptive research development areas where, in a perfect non‐capitalistic context, we should be putting our money and effort, and not in bigger and ‘better’ accelerators that may only cost us markedly more to give therapeutically effective doses similar to what is being delivered by thousands of already existing linear accelerators in the United States.

## 4 REBUTTAL

### 4.1 Eleanor Blakely, PhD; Bruce Faddegon, PhD; Christopher Tinkle, MD, PhD

The following is a point‐by‐point rebuttal to the position of the “against side.”

1. There is an enormous potential for benefit from CIRT. Given the known radiobiologic and dosimetric advantages, the potential patient pool will be much larger than that thought to possibly benefit from protons, just as we have seen with significantly higher utilization of IMRT over 3DCRT. The “new biology” recognized by the “against side” justifies the identification of additional patients who may benefit from the unique mechanisms of action of CIRT to mitigate multifactorial tumor radioresistance (see ref. 24). Additionally, any CIRT facility within the United States would necessitate a location near a large population center, which would help ensure adequate patient numbers to maintain operations.
2. In stark contrast to the early proton experience, the hard lessons learned in establishing proton therapy in the United States would help ensure more fiscally and scientifically sound efforts by the CIRT community. A new CIRT facility in the United States would be the center point for the design, implementation, and evaluation of a multi‐site, multi‐national effort of rationally designed clinical trials to establish the utility of CIRT. This would be modelled on the current successful collaborative clinical trial efforts with international CIRT sites. The US government could support R&D at the facility in the form of NCI‐sponsored clinical trials and grants to explore the engineering, physics, and biology of CIRT. The NCI has already demonstrated its support through funding of a pair of P20 planning grants to leverage CIRT R&D in a new facility, when it is built, and a trial of pancreatic cancer with a CIRT arm.^48^
3. The difficulty in convincing insurers to reimburse treatment is a common problem for new technology and one which should not dictate the next generation of radiotherapy techniques. Putting the patient first means implementing promising new technology that includes R&D to make it cost‐effective, guided by high‐level site‐specific evidence. We are not far from CIRT becoming cost‐effective: for chordoma, there are already published reports of this even with currently available technology.^68^, ^69^
4. Here we address the “against sides” perception of the lack of strong support for a clinical replacement to the pioneering CIRT program at Berkeley. The promising results from Berkeley^29^, ^30^, ^31^, ^32^, ^33^, ^34^, ^35^, ^36^, ^37^ provided crucial support for construction of CIRT facilities in several countries, which has further led to promising data from completed well‐designed clinical trials showing significant clinical advantages, as shown earlier. These early phase studies should be sufficient clinical evidence to justify a CIRT center in the United States, a point we address shortly. There is also renewed interest at the NCI to support CIRT, as mentioned earlier.
5. We agree that there are advantages to establishing multiple centers, but this does not rule out starting with a single center. New technology must be implemented in at least one center to establish its effectiveness. Since it will take longer to accrue patients for clinical trials with only one center, we need to start now. The first several proton centers established in the United States were instrumental in leading to additional centers, as will be the case with CIRT.
6. Experience with neutrons was largely seen as a negative. In fact, the neutron RBE is quite effective, although the physics is suboptimal for deeper lesions, and the need for boron drug specificity for tumor has been a limitation for boron neutron capture therapy (BNCT). However, new accelerator‐based sources of neutrons are being evaluated in clinical trials, and despite the problems, BNCT experts in Finland are curing resistant tumors.^70^
7. Several issues with the current state of CIRT technology were considered as arguments against the proposal, but the reverse is true. A US CIRT treatment and research center will lead to rapid and cost‐effective technology advances. For example, range uncertainty mitigation and motion management are solvable problems. Although an SOBP has lower RBE than the Bragg peak, CIRT will still have a higher peak‐to‐plateau ratio in RBE‐weighted dose than protons. Regarding complexities of beam precision, one can always smudge the dose distribution, but one cannot improve intrinsic beam precision, an undeniable advantage of carbon over protons.
8. Directing investment away from CIRT into potential "game‐changing" new investigations as the “against side” proposes is counter‐productive. Emerging technologies such as immunotherapy, MR‐linked therapy,^71^ and FLASH radiotherapy all may well show enhanced novel effects with particle therapy. Having a US CIRT center would facilitate investigations into how heavy charged particles may complement and even amplify their effects.

Finally, we review recent outcomes of patients with adenoid cystic carcinoma (ACC) to raise the provocative question of how much data are needed to accept the utility of CIRT. Preclinical radiobiology studies and clinical investigations with charged particles have demonstrated favorable outcomes compared to photons, particularly with regard to toxicities, of this rare, yet highly aggressive neoplasm. Based on these early studies, photon therapy was combined with protons, resulting in 5‐year disease‐free survival and overall survival (OS) rates of 56% and 77%, respectively.^72^ CIRT alone has resulted in 5‐year OS rate of 68%,^73^ whereas a recent Japanese study reported a 5‐year OS of 70% with acceptable toxicities. Additional comparisons of proton and CIRT have demonstrated no significant differences in OS, but toxicities were increased in those treated with protons.^74^ In a 15‐year follow‐up study, IMRT with a raster‐scanned carbon ion boost yielded good tumor control with moderate toxicities.^39^ The Italians compared IMRT to charged particles, yet differences in patient, treatment, and evaluation metrics limited conclusions.^75^ A subsequent Japanese study of 289 patients with ACC treated with CIRT alone at four different facilities yielded a 5‐year OS of 74% with few significant toxicities.^42^ Most recently, a 2‐year OS of 100% in patients treated with CIRT has been reported.^45^ Thus, the significant improvements in patient outcomes over the last decade are due, at least in part, to the use and technical advancement of CIRT. While this may not constitute Level 1 evidence, the data are compelling and may justify a more rapid alternative to the requisite randomized phase III trial to establish clinical utility. We believe the evidence for CIRT is already solid and that we need a center in the United States so our citizens can benefit from the same effective treatment available to citizens of other countries.

### 4.2 Charles Bloch, PhD; Michael Dominello, DO; Robert Griffin, PhD

Drs. Faddegon, Tinkle, and Blakely have done an excellent job describing the status of carbon ion radiation therapy (CIRT). Unfortunately, the strongest argument against building such a facility comes from their introduction. As they state, much of the research has been done a long time ago (1967) at LBNL. As they themselves point out, that program was stopped due to cost, which remains just as much an issue today as it was then. As we have already stated, proton facilities struggle with cost issues, and our opponents state as much in their opening argument.

They also point out the absence of high‐level clinical evidence for CIRT. More accurately, it is a lack of high‐level compelling evidence even after treatment of over 27,000 CIRT patients as of 2017.^76^ This is the same basic issue proton therapy struggles with to obtain coverage and reimbursement from insurers in the United States. While physicists and physicians point to the “obvious” advantage of the Bragg peak and treatment planning comparisons showing integral dose reductions of a factor of 2 or more, the question remains: If the physics has such a dramatic advantage, why is the clinical advantage so elusive? In total, 30 proton therapy centers in the US struggle to provide compelling clinical evidence for the advantage over x rays; we surmise that a single CIRT is not going to be able to show a greater advantage compared to what is already questionable with protons.

Our opponents also mention the current developments in proton/particle beam therapy (pencil beam scanning and reduction in range uncertainty). Certainly, these would be utilized in any new CIRT, but just as in proton therapy, they will only help CIRT keep up with the many advances in x‐ray therapy. They are not the silver bullet.

Indeed, as our opponents show there has been a great deal of study of the radiobiologic effects of ion therapy. Unfortunately, promising in‐vivo research results often do not translate to the hoped‐for clinical results. Our colleagues have already admitted that there is a lack of high‐level evidence for CIRT. Radiobiology studies would be useful to determine how to best exploit CIRT. These studies are not a reason to build a CIRT. One needs more evidence than that. Although the radiobiology of carbon ions in tumor suggests that improved tumor control is possible compared to photons, the lack of increase in therapeutic ratio is the major hurdle that has not been met. The increasing ability of linear accelerators to conformally treat in 3D at very high doses and low normal tissue doses, combined with new developments suggesting that photons can be used to obtain an even greater therapeutic index using ultra‐high‐dose rates (FLASH), means that carbon ions will have a very hard time justifying their use.

It follows that the clinical evidence cited by our opponents is weak, even as they describe it. The early US data are not a clinical trial. The Japanese and German data are only phase I/II trials; no phase III trials are completed. Furthermore, our opponents mention the results are “encouraging” for “tumors which are often resistant to conventional photon irradiation.” No advantage over more affordable proton therapy is suggested let alone shown. And finally, the authors point out that one cannot draw conclusions from trials which exclude some populations as the response can vary depending on the population studied.

In their conclusion, the authors state that CIRT would have a superior dose distribution compared to protons. We disagree. Carbon ions undergo fragmentation, sending dose beyond the Bragg peak. The Bragg peak itself is positioned beyond the tumor to ensure tumor coverage putting the highest LET and highest RBE in normal tissue which, as mentioned above, will remain the greatest obstacle and practical barrier to obtaining improved patient outcomes. Protons clearly have a superior dose distribution over photons yet still struggle to show a clinical advantage. The difference between the carbon dose distribution and that of protons is much smaller by comparison so one cannot imagine it will have a markedly different or significant impact.

Finally, the authors argue that a CIRT will employ the best technology, have a decades long life (to reduce cost), and use a reduced number of fractions. These are incompatible objectives. To show any advantage over current treatments, the best technology will have to be updated continuously. It will not be the best technology in 5 yr let alone 10 or 20 or 30 yr. And the ideal fractionation has already been stated as one of the goals of CIRT investigation, yet they realize they will be limited to hypofractionation for cost reduction. In conclusion, a single CIRT facility in the United States is unlikely to establish high‐level compelling evidence of a viable advantage over existing treatment options and we maintain that it will not be a worthwhile investment in this country for the foreseeable future.

## CONFLICTS OF INTEREST

The authors declare no conflicts of interest.

## DISCLAIMER

The views and opinions of authors expressed herein do not necessarily state or reflect those of US Government or any agency thereof or the Regents of the University of California.
